# Inequalities in self-rated health among 45+ year-olds in Almaty, Kazakhstan: a cross-sectional study

**DOI:** 10.1186/1471-2458-13-654

**Published:** 2013-07-15

**Authors:** Akmaral K Abikulova, Kazbek A Tulebaev, Aikan A Akanov, Botagoz S Turdalieva, Sundetgali B Kalmahanov, Ainur B Kumar, Aigulsum K Izekenova, Bakhyt A Mussaeva, Andrej M Grjibovski

**Affiliations:** 1 School of Public Health, Kazakh National Medical University, Almaty, Kazakhstan; 2 Kazakh National Medical University, Almaty, Kazakhstan; 3 International School of Public Health, Northern State Medical University, Arkhangelsk, Russia; 4 Department of International Public Health, Norwegian Institute of Public Health, Postbox 4404, Nydalen, 0403, Oslo, Norway

**Keywords:** Central Asia, Kazakhstan, Self-rated health, Determinants, Socio-demographic

## Abstract

**Background:**

Self-rated health (SRH) has been widely studied to assess health inequalities in both developed and developing countries. However, no studies have been performed in Central Asia. The aim of the study was to assess gender-, ethnic-, and social inequalities in SRH in Almaty, Kazakhstan.

**Methods:**

Altogether, 1500 randomly selected adults aged 45 years or older were invited to participate in a cross-sectional study and 1199 agreed (response rate 80%). SRH was classified as poor, satisfactory, good and excellent. Multinomial logistic regression was applied to study associations between SRH and socio-demographic characteristics. Crude and adjusted odds ratios (OR) for poor vs. good and for satisfactory vs. good health were calculated with 95% confidence intervals (CI).

**Results:**

Altogether, poor, satisfactory, good and excellent health was reported by 11.8%, 53.7%, 31.0% and 3.2% of the responders, respectively. Clear gradients in SRH were observed by age, education and self-reported material deprivation in both crude and adjusted analyses. Women were more likely to report poor (OR = 1.9, 95% CI: 1.2-3.1) or satisfactory (OR = 1.6, 95% CI: 1.2-2.1) than good health. Ethnic Russians and unmarried participants had greater odds for poor vs. good health (OR = 2.3, 95% CI: 1.5-3.7 and OR = 4.0, 95% CI: 2.7-6.1, respectively) and for satisfactory vs. good health (OR = 1.4, 95% CI: 1.1-1.9 and OR = 1.9, 95% CI: 1.4-2.5, respectively) in crude analysis, but the estimates were reduced to non-significant levels after adjustment. Unemployed and pensioners were less likely to report good health than white-collar workers while no difference in SRH was observed between white- and blue-collar workers.

**Conclusion:**

Considerable levels of inequalities in SRH by age, gender, education and particularly self-reported material deprivation, but not by ethnicity or marital status were found in Almaty, Kazakhstan. Further research is warranted to identify the factors behind the observed associations in Kazakhstan.

## Background

Self-rated health (SRH) has been widely studied to assess health inequalities in both developed and developing countries. SRH is considered to be a simple, valid and reliable indicator and an applicable tool for use in population-based epidemiological studies as a predictor of overall morbidity [1] and mortality among elderly [2] and general population [3].

Former republics of the Soviet Union have experienced a profound economic and social crisis during the 1990s, which was accompanied by increase in income and health inequalities. Interestingly, while economic indicators show rapid economic growth in most of these countries during 2000s, they were not accompanied by either reduction of inequalities or considerable improvements in population health [4].

SRH varies considerably both between and within countries [5]. Correlates of SHR can be classified into several domains: socio-demographic, health conditions, psychological factors, social support and health behaviors [6]. Several studies have shown social class and age to be independent predictors of SRH [6-8]. Although gender variations in SRH have not been a universal finding [9], women are consistently more likely to report poor health compared to men in developing [7] countries and in countries of the former Soviet Union [10]. Moreover, similarly to Western societies, in former Communist countries of Eastern Europe, education and material deprivation are strongly associated with SHR [11].

In spite of a considerable volume of evidence on SRH and its determinants from Western societies and European transitional economies, we could not identify a single study from any of the former Soviet republics located in Central Asia in international peer-reviewed literature.

Kazakhstan is the second largest country among the former republics of the Soviet Union with rapidly developing economy with annual growth of 8.9% in 1999–2003. GDP per capita in increased from 2,056 USD in 2003 to 8,326 USD in 2011, but is still below the world average (8,985 USD). Kazakhstan is a multiethnic state with a total population of 16.4 million (2011). The majority of the population are Kazakhs (63.1%) and the most populous ethnic minority are Russians (23.7%). Life expectancy in Kazakhstan is among the lowest in the European WHO region with one of the greatest gender gaps in the world: 63.6 years for men and 73.5 years for women in 2009 [4]. However, income distribution is substantially more equal than in other countries of the former Soviet Union. Thus, Gini coefficient in Kazakhstan was 24.8 in 2005, while in Russia and Ukraine the corresponding numbers were 35.9 and 33.8, respectively. Whether lower level of income inequalities are reflected by lower level of health inequalities in Kazakhstan remains unknown.

The aim of the study was to assess gender-, ethnic-, and social inequalities in SRH among adults aged 40-years or older in the second largest city of Central Asia–Almaty, Kazakhstan.

## Methods

This population-based cross-sectional study was performed in Almaty (Alma-Ata until 1993)–the largest city and the former capital of Kazakhstan. In spite of the fact that the capital of Kazakhstan moved to Astana in 1997, Almaty remains the wealthiest city in the country with the highest average income and high levels of inequalities.

A total of 1500 individuals aged 45 years or older residing in the Almalinski district of Almaty were selected at random from the total population of the selected age group of the district and invited to participate in the study. This district was selected because it is representative of the total population of the city in terms of age and gender structure. This study is a part of a larger cohort study on healthy aging in Almaty, therefore only those aged 45 years or older were sampled. Altogether, 1199 agreed to participate in the study when they contacted by the interviewers (response rate 80%). Trained interviewers visited study participants in their homes and filled out a 160-items questionnaire on health-related issues. For the purpose of this paper, only questions related to SRH and its socio-demographic correlates were used because of their acceptable face validity and potential comparability with international studies.

SRH status is based on one question and categorized as poor, satisfactory, good and excellent. Age of the participants was classified as 45–54, 55–64 years, 65–74 years and 75 years or older. Education was coded as secondary or less, vocational and higher. By ethnic background, the participants were classified as Kazakhs, Russians or others/unknown. Marital status was coded as married or unmarried. The latter category included single, co-habiting, divorced and widowed. By occupation, the participants were grouped as blue-collar workers, white-collar workers, unemployed, pensioners and other/unknown. The following categories reflecting household material deprivation: not enough even to by food (category 1); enough money to buy food, but not new clothes (category 2); enough money to buy food and clothes (category 3); enough money to buy more expensive items (category 4), and unknown.

Bivariate analyses were performed using Pearson’s chi-squared tests. Independent associations between SRH and studied socio-demographic correlates were assessed by multinomial logistic regression analysis with good self-rated health as a reference. Cases with missing data on SRH, age, education or marital status (n = 25 or 2.1%) were excluded from multivariable analyses. Crude and adjusted odds ratios (OR) for poor vs. good health and for satisfactory vs. good health with 95% confidence intervals (CI) were calculated. Trends for age, education, and material deprivation were assessed by including these variables into regression models as continuous variables. All analyses were performed using SPSS v. 16.0 (SPSS Inc, Chicago, Il, USA).

The study was approved by the ethical committee of the Kazakh National Medical University in 2011.

## Results

Most of the study participants (53.7%) rated their health as satisfactory while only 3.2% reported having excellent health. Because of small number of cases with excellent health, this category was merged together with good SRH in further analyses. Most of the responders were Russians, had higher education, were women, pensioners, married and belonged to the age-group 45–54 years. By material deprivation, nearly every tenth respondent reported having not enough money to buy food items while 19.8% of respondents did not indicate their level of material deprivation (Table 1).

**Table 1:** Background characteristics of the sample

**Characteristic**	**N**	**%**
Age, years		
45–54	416	34.7
55–64	347	29.1
65–74	270	22.7
75+	158	13.3
Unknown	8	0.7
Gender		
Male	505	42.1
Female	694	57.9
Ethnic background		
Kazakh	400	33.4
Russian	644	53.7
Other/Unknown	155	12.9
Education		
Secondary or less	347	28.9
Vocational	283	23.6
Higher	569	47.5
Occupation		
Blue-collar	261	21.8
White-collar	194	16.2
Unemployed	143	11.9
Pensioner	567	47.3
Other/unknown	34	2.8
Marital status		
Married	832	69.4
Unmarried	354	29.5
Unknown	13	1.1
Self-reported material deprivation		
Category 1	162	13.5
Category 2	218	18.2
Category 3	285	23.8
Category 4	296	24.7
Unknown	238	19.8
Self-rated health		
Poor	141	11.8
Satisfactory	644	53.7
Good	372	31.0
Excellent	38	3.2
Unknown	4	0.4
Total	1199	100.0

We observed clear variations of SHR across all socio-demographic variables in crude analysis. The most pronounced inequalities in SRH were found by material deprivation: While only 4.1% of respondents from the most privileged category reported poor health, the corresponding proportion among the least privileged was 24.8%. Similarly, more than a half of those with the lowest level of material deprivation reported good health while only 11.2% of those with not enough money to buy food items had good SRH. Clear positive gradient between education and SRH was observed, while the association between SRH and age was inverse. Married responders were more likely to report better health than unmarried. Women had poorer SRH than men while Kazakhs reported better health than ethnic Russians (Table 2).

**Table 2:** Self-rated health across socio-demographic characteristics of the sample (n = 1174)

**Characteristic**	**Poor**	**Satisfactory**	**Good**^**a**^	**P**^**b**^
Age, years				<0.001
45–54	20 (4.9)	189 (46.3)	199 (48.8)	
55–64	29 (8.5)	184 (53.8)	129 (37.7)	
65–74	54 (20.2)	154 (57.7)	59 (22.1)	
75+	35 (22.3)	106 (67.5)	16 (10.2)	
Gender				<0.001
Male	42 (8.4)	239 (47.9)	218 (43.7)	
Female	96 (14.2)	394 (58.4)	185 (27.4)	
Ethnic background				0.002
Kazakh	32 (8.2)	201 (51.7)	156 (40.1)	
Russian	92 (14.5)	353 (55.5)	191 (30.0)	
Other/Unknown	14 (9.4)	59 (57.0)	76 (37.6)	
Education				<0.001
Secondary or less	65 (19.1)	197 (58.6)	74 (22.0)	
Vocational	43 (15.5)	155 (55.8)	80 (28.8)	
Higher	30 (5.4)	281 (50.2)	249 (44.5)	
Occupation				<0.001
Blue-collar	9 (3.5)	114 (44.7)	132 (51.8)	
White-collar	9 (4.7)	68 (35.8)	113 (59.5)	
Unemployed	13 (9.6)	83 (61.0)	40 (29.4)	
Pensioner	102 (18.2)	355 (63.4)	103 (18.4)	
Other/Unknown	5 (15.2)	13 (39.4)	15 (45.5)	
Marital status				<0.001
Married	69 (8.4)	431 (52.4)	323 (39.2)	
Unmarried	69 (19.7)	202 (57.5)	80 (22.8)	
Self-reported material deprivation				<0.001
Category 1	40 (24.8)	103 (64.0)	18 (11.2)	
Category 2	39 (18.1)	141 (65.6)	35 (16.3)	
Category 3	26 (9.3)	159 (56.8)	95 (33.9)	
Category 4	12 (4.1)	126 (43.3)	153 (52.6)	
Unknown	201 (9.3)	104 (45.8)	102 (44.9)	
Total (n = 1174)	138 (11.8)	633 (53.9)	403 (34.3)	

^a^Good and excellent combined.

^b^Compared using Pearson’s chi-squared test.

After adjustment for all studied variables, variations in SRH across ethnic backgrounds and marital status were reduced to non-significant levels (Table 3). However, associations between SRH and age, gender, education and material deprivation remained significant independently of other studied factors. Social variations in SRH by material deprivation were the most pronounced even after adjustment for other variables. Those who reported poor health had 20.0 times greater odds for belonging to the poorest category compared to those who reported good health, while those who reported satisfactory health had corresponding odds ratio of 6.3. Clear social gradient by material deprivation was observed for reporting both poor (p for trend <0.001) and satisfactory (p for trend <0.001) health. Similarly to most other studies, older participants had greater odds of reporting poor than good health (p for trend <0.001), but only those older than 75 years were more likely to report satisfactory than good health. Participants with other education than higher were more likely to report poor than good health, although the association between education and reporting satisfactory health vs. good health did not reach the level of statistical significance. Pensioners had on average three times greater odds to report poor than good health and four times greater odds to report satisfactory than good health. Unemployed responders had more than twice as high odds for reporting satisfactory than good health (Table 3).

**Table 3:** Results of the multinomial logistic regression: crude and adjusted odds ratios (OR) with 95% confidence intervals (n = 1174)

**Characteristic**	**Poor vs. Good**^**a**^		**Satisfactory vs. Good**^**a**^	
	**Crude OR**	**Adjusted OR**^**b**^	**Crude OR**	**Adjusted OR**^**b**^
Age, years				
45–54	Reference	Reference	Reference	Reference
55–64	2.2 (1.2-4.1)	1.6 (0.8-3.3)	1.5 (1.1-2.0)	1.1 (0.7-1.5)
65–74	9.1 (5.0-16.4)	2.6 (1.1-6.3)	2.7 (1.9-3.9)	0.9 (0.5-1.6)
75+	21.8 (10.3-46.0)	6.4 (2.3-17.9)	7.0 (4.0-12.2)	2.6 (1.3-5.4)
Gender				
Male	Reference	Reference	Reference	Reference
Female	2.7 (1.8-4.1)	1.9 (1.2-3.1)	1.9 (1.5-2.5)	1.6 (1.2-2.1)
Ethnic background				
Kazakh	Reference	Reference	Reference	Reference
Russian	2.3 (1.5-3.7)	1.0 (0.6-1.7)	1.4 (1.1-1.9)	1.0 (0.7-1.3)
Other/Unknown	1.2 (0.6-2.5)	0.5 (0.2-1.2)	1.1 (0.7-1.6)	0.7 (0.4-1.1)
Education				
Secondary or less	7.3 (4.4-12.1)	3.5 (1.9-6.2)	2.4 (1.7-3.2)	1.5 (1.0-2.1)
Vocational	4.5 (2.6-7.6)	3.2 (1.8-5.8)	1.7 (1.3-2.4)	1.4 (0.9-2.0)
Higher	Reference	Reference	Reference	Reference
Occupation				
Blue-collar	0.9 (0.3-2.2)	0.6 (0.2-1.6)	1.4 (1.0-2.7)^c^	1.2 (0.8-1.8)
White-collar	Reference	Reference	Reference	Reference
Unemployed	4.1 (1.6-10.3)	1.8 (0.7-5.0)	3.5 (2.1-5.6)	2.3 (1.4-3.9)
Pensioner	12.4 (6.0-25.9)	3.2 (1.3-8.0)	5.7 (3.9-8.3)	4.0 (2.4-6.6)
Other/Unknown	4.2 (1.2-14.2)	3.4 (0.9-12.9)	1.4 (0.7-3.2)	1.3 (0.6-3.1)
Marital status				
Married	Reference	Reference	Reference	Reference
Unmarried	4.0 (2.7-6.1)	1.6 (1.0-2.7)^c^	1.9 (1.4-2.5)	1.1 (0.8-1.5)
Self-reported material deprivation				
Category 1	28.3 (12.6-63.6)	20.0 (8.3-47.9)	6.9 (4.0-12.1)	6.3 (3.5-11.4)
Category 2	14.2 (6.8-29.9)	12.3 (5.5-27.4)	4.9 (3.2-7.6)	4.7 (2.9-7.6)
Category 3	3.5 (1.7-7.2)	4.4 (2.0-9.7)	2.0 (1.4-2.9)	2.5 (1.7-3.6)
Category 4	Reference	Reference	Reference	Reference
Unknown	2.6 (1.3-5.6)	3.3 (1.5-7.4)	1.2 (0.9-1.8)	1.5 (1.0-2.2)^c^

^a^Good and excellent combined.

^b^Adjusted for all variables in the table.

^c^Confidence intervals include 1.0.

## Discussion

The results of this first to our knowledge study on SRH in Central Asian republics of the former Soviet Union suggest that nearly two thirds the population aged 45 years and older rates their health as satisfactory or poor while excellent health was reported by only 3.2% of the responders. The proportion of those who rated their health as lower than good in our sample is greater than in comparable age-groups in Syria [6], but also exceeds the prevalence of poorer than good health among Australian, Japanese and American in older age-group [5,12]. However, direct comparisons are difficult to make because different age groups were studied in different studies. Given that SRH is considered to be a simple, valid and reliable health indicator for use in population-based epidemiological studies as a predictor of overall morbidity and mortality [1-3], one may speculate that the general health status of the population in Almaty is poorer than in most developed countries, which is reflected by the fact that Kazakhstan has one of the lowest life expectancies in the European WHO region [4]. At the same time the prevalence of poor SRH observed in this study is lower not only than in Russia and Ukraine [10,13], but also lower than in Estonia, Latvia, Lithuania, Poland and Hungary in the mid-1990s [11], especially given the fact that study participants in these countries were considerably younger than in our sample where the youngest participants were 45 years old. Compared to the latest data on the prevalence of poor SRH measured in 2003–2004 in 13 former communist European countries, our estimates are close to what was observed in Slovenia and are more favorable than the results obtained in all other countries, where the prevalence of poor SRH ranged from 10.4% in Slovenia and 24.3% in Ukraine [14], but the sample in these countries included participants 18 years and older. Thus, although the prevalence of poor SRH in Almaty is considerably higher than in developing countries, our findings suggest that poor SRH is less prevalent than in European post-communist economies during the time of transition. However, direct comparisons are not possible given differences in age-groups and years when the studies were performed.

Compared to other studies on inequalities in SRH, the observed variations in SRH by the level of material deprivation are greater than in most other studies (Table 3). Differences between the results of our study and other studies may be partly explained by the differences between the measures of material deprivation. Given that data on income are difficult to obtain in countries of the former Soviet Union and that respondents tend to underestimate their real income [15], we used the self-reported measure of material deprivation that reflects the purchase power of the households which we consider as a more valid tool for use in this population. In other studies, measures of societal position [10], social class [8] or material deprivation [11] or socio-economic status scores [6] were used complicating comparisons of our findings with results of these studies. Nevertheless, the finding that people belonging to the least privileged category had 20.0 times greater odds for reporting poor than good health compared to those without material problems is a matter of concern. However, given that odds ratios overestimate the effect if the outcome is common as in this case, the point estimates obtained in our study should be interpreted with due caution. Moreover, the proportion of the participants who does not have enough money to purchase food was 13.5% and nearly a third of the respondents do not have enough money to buy clothes (Table 1). This reflects poor material status of the considerable proportion of the population even in the wealthiest city in Kazakhstan and may be one of the factors behind poor health status in the country in general.

As in numerous studies from other countries, we found an inverse relationship between age and SRH. However, the gradient by age was more pronounced between poor and good health, while the only those who were 75 years or older were more likely to report satisfactory than good health in our sample (Table 3). Associations between age and poor health are well-known and can be explained by poorer objective health status and greater number of chronic diseases among the elderly [16].

In addition to pronounced variations in SRH across categories of material deprivation we also observed a more pronounced variation by education compared to the two other former Soviet Republics of Russia and Ukraine [10,11]. However, the differences between the findings can be partly attributed to the differences in the data analysis and adjustment factors. While in the Ukrainian and Russian studies the odds ratios were calculated for good vs. less than good [10] or by poor vs. better than poor [14], we applied multinomial regression to further explore the associations that might be associated with the likelihood of reporting good vs. bad and good vs. satisfactory health. As expected more pronounced differences by education were observed between poor and good health, while the differences between the group with the lowest and the highest educational levels also reached the level of statistical significance even after adjustment for all other studied factors. These results may reflect healthier lifestyle choices and better general health among those who are better educated independently of age, material deprivation, gender and occupation, which were also associated with SRH.

Interestingly, we did not find the differences in SRH by occupation except for the group of pensioners. This may reflect the situation in Kazakhstan in other former Soviet Republics, where several white-collar occupations such as medical professionals and teachers are among the least paid. There is a clear distinction between white- and blue-collar occupations in Kazakhstan, so the findings cannot be explained by ambiguity of the question. However, while no differences were found by employment status in Ukraine [10], unemployed in Kazakhstan were more likely to report satisfactory than good health, although no differences between white-collar workers and unemployed were observed in the odds of reporting poor vs. good health.

Kazakhstan in a multiethnic state with declared equality of all ethnic groups residing on its territory. Our findings suggest that although ethnic Russians are more likely to report poor or satisfactory health than ethnic Kazakhs, these differences seem to be attributed to other socio-demographic factors, but not ethnic background per se. However, given that we observed ethnic differences in crude analysis, but not after adjustment for other factors suggests considerable differences between ethnic Kazakhs and ethnic Russians in other variables, particularly in material factors. Moreover, given that Russians constitute 53.7% of the sample while in the total population of Almaty their share is 33.0%, the overall prevalence of poor or satisfactory SRH in the study is likely to be overestimated.

Gender variations in SRH have been in many countries; however, the direction of association varies across the settings. Women are more likely to report poor health than men in most of the countries including both egalitarian societies [17] and countries with high levels of inequalities [5-7,10]. However, no differences have been reported in Australia and Japan while Korean men were more likely than Korean women to report poor health [5]. Thus, variation in SRH across genders is not a universal phenomenon [9] and in Kazakhstan, as in most other countries, women tend to report poor health more often than men. Whether our finding reflects poorer health of Kazakhstani women or reporting bias requires further research.

Marital status is not universally associated with SRH in different settings. While in the USA and Australian living with a partner was associated with poorer SRH, the opposite was observed in Korea and no association was found in Japan in age-group comparable to ours [5]. Previous studies from Eastern Europe and from the European part of the former Soviet Union have shown no association between SRH and marital status, which is in line with our findings. However, there is evidence that unmarried women are more likely to report poor health than married in some Asian countries [6]. However, although situated in Asia, Kazakhstan as a former Soviet republic and its largest city where the study was performed is closer to the European tradition of non-discrimination of women by marital status. Co-habiting or common marriages have become more popular in recent years in urban settings of Kazakhstan. These women were classified as unmarried in this study, which might decrease the effect of being single on the studied outcome. However, the proportion of common marriages is about 15% in the studied age-group and thus has limited effect on the estimates. We replicated our analyses separately by gender and did not find associations between marital status and SRH among either women or men.

The results of this study should be interpreted with caution due to its potential limitations. First, the data were collected from one district in the former capital of Almaty. Although the sample is representative by age- and gender structure, we do not have information on whether our sample is representative by other characteristics of the total population of Almaty. Given its central location one may speculate that the participant of the study is better educated and wealthier than the reference population. Moreover, given that Almaty is the former capital of Kazakhstan and is still the wealthiest city, the findings should not be generalized to Kazakhstan or other Central Asian countries, where populations are far less privileged than in Almaty. The response rate was 80%. Non-responders are often more likely to be younger and to be men, which was not the case in this study, however, non-responders might be more likely to have better health than responders because they were not at home at the time of the study suggesting the prevalence of poor SRH may be slightly overestimated. However, we do not have information about the health status of non-responders and thus, our speculation should be read with caution.

Although SRH is considered to be a measure of health with acceptable validity for large epidemiological studies [1-3] and strong association with mortality [3], lack of objective data on physical and mental conditions is another limitation of the study. However, the main aim of the study was not to assess health status per se, but inequalities in SRH across several socio-demographic variables and for this aim SRH seems to be an appropriate outcomes measure, which was used in many countries. Nevertheless, although our findings can reflect social variations in SRH in Kazakhstan, direct comparisons of the associations between socio-demographic factors and SRH are difficult, because most of the studies use a 5-point scale while we used a 4-point scale, which was further reduced to a 3-point scale for the analysis.

While the variables used in this study, such as age, gender, marital status, ethnic background, education, occupation and material deprivation category based on purchase power are relatively easy to measure and can be considered as valid, the validity of SRH and differential reporting can be further discussed. People with lower social positions may report poorer health than their objective health while better-off may overestimate their health [18] contributing to greater inequalities in SRH than in objective health. Moreover, women and Hispanics are known to incorporate more mental health into reported health [19]. Whether this differential reporting by the level of material deprivation, gender and ethnicity in our sample or in Kazakhstan in general is the case remains unknown, we suggest interpreting our results carefully du to this potential limitation.

Another limitation of the study is its cross-sectional design, which does not allow studying causative relationships. However, most of the studied factors are known to precede the measure of health, such as age, gender, ethic background and education, association between SRH, material deprivation and occupation may be sensitive to health selection bias: low income, for example, may both cause poor health and be a result of it, although it has been suggested that this bias is a minor component of health inequalities [20].

Societal measures of well-being, such as corruption and GDP, but not income inequalities in the population level were associated with poor SRH in countries of Central and Eastern Europe [11,14] partly supporting our findings which show that despite lower levels of income inequalities in Kazakhstan compared to Russia and Ukraine, the level of inequalities in SRH seem to be even greater than in these countries. Further research is needed to elucidate the factors that may explain the observed inequalities in SRH in Almaty, Kazakhstan.

Given the limitations of the study, particularly, the self-reported nature of the data and different classification of SRH, the results should be interpreted and compared with results from other countries with due caution Moreover, given that the sample is representative only to the city of Almaty and only by age and gender structure, we do not recommend generalizing the findings to other regions of Kazakhstan particularly to rural areas.

## Conclusions

Considerable levels of inequalities in SRH by age, gender, education and particularly material deprivation, but not by ethnicity or marital status were found in Almaty, Kazakhstan. The observed differences seem to be even more pronounced than in Russia and Ukraine–countries, which share with Kazakhstan their Soviet past, but have greater levels of income inequalities at present. Further research is warranted to identify the factors behind the observed inequalities in SRH in Kazakhstan.

## Competing interests

The authors do not have any conflicts of interest.

## Authors’ contributions

All authors participated in designing the study. All authors except AMG participated in data collection. AMG analyzed the data. AMG and AKA drafted the manuscript. All authors approved the final version of the paper. All authors read and approved the final manuscript.

## Pre-publication history

The pre-publication history for this paper can be accessed here:

http://www.biomedcentral.com/1471-2458/13/654/prepub
